# *Celastrus orbiculatus* extract inhibits the migration and invasion of human glioblastoma cells in vitro

**DOI:** 10.1186/s12906-016-1232-8

**Published:** 2016-10-06

**Authors:** Hao Gu, Jun Feng, Haibo Wang, Yayun Qian, Lin Yang, Jue Chen, Feng Jin, Youyang Shi, Songhua Lu, Yangqing Liu

**Affiliations:** 1 Yangzhou University-Yangzhou Cancer Research Institute, Yangzhou, 225009 China; 2 The Key Laboratory of Cancer Prevention and Treatment of Yangzhou University, Yangzhou, 225009 China; 3 Medical&Pharmaceutical Institute of Yangzhou University, Yangzhou, 225009 China

**Keywords:** *Celastrus orbiculatus*, Glioblastoma, EMT, Invasion, Migration, Actin

## Abstract

**Background:**

Gliomas are highly aggressive tumors of the nervous system, and current treatments fail to improve patient survival. To identify substances that can be used as treatments for gliomas, we examined the effect of *Celastrus orbiculatus* extract (COE) on the invasion and migration of human glioblastoma U87 and U251 cells in vitro*.*

**Methods:**

The effects of COE on cell viability and adhesion were tested using the 3-(4,5-dimethyl-2-thiazolyl)-2,5-diphenyl-2-H-tetrazolium bromide assay and cell adhesion assay, respectively. The effects of COE on cell migration and invasion were assessed by a wound-healing assay and transwell migration and invasion assays. The effects of COE on the expression of epithelial-mesenchymal transition (EMT)-related proteins and matrix metalloproteinases (MMPs) were evaluated using western blot and gelatin zymography, respectively. Finally, the effect of COE on actin assembly was observed using phalloidin-tetramethylrhodamine isothiocyanate labeling and confocal laser scanning microscopy.

**Results:**

We found that COE inhibited the adhesion, migration, and invasion of U87 and U251 cells in a dose-dependent manner. COE reduced N-cadherin and vimentin expression, increased E-cadherin expression, and reduced MMP-2 and MMP-9 expression in U87 and U251 cells. Furthermore, COE inhibited actin assembly in U87 and U251 cells.

**Conclusions:**

COE attenuates EMT, MMP expression, and actin assembly in human glioblastoma cells, thereby inhibiting their adhesion, migration, and invasion in vitro.

## Background

Gliomas are primary brain tumors derived from brain and spinal glial cells. Because gliomas are highly aggressive, they represent 80 % of all malignant tumors of the human nervous system and are associated with a high rate of recurrence [1]. Current treatments for gliomas include surgery, radiotherapy, and chemotherapy, but these treatments do not improve patient prognosis and have major side effects on brain tissue, resulting in median survival duration of only 9 to 10 months [2]. The current treatments do not completely remove tumor cells generated by the glioblastoma, invasion and migration major leading death [3]. Therefore, it is imperative to develop drugs that can effectively inhibit the invasion of glioblastoma cells to improve patient survival. Our previous studies show that COE inhibits the invasion and migration of tumor cells in liver cancer [4], gastric cancer [5], and colorectal cancer [6]. Here, using human glioblastoma U87 and U251 cell lines, we examined the effects of COE on the invasion and migration of glioblastoma cells and their underlying molecular mechanisms.

## Methods

### Cell lines and reagents

Human glioblastoma U251 and human malignant glioblastoma U87 cell lines (Shanghai Cell Bank of the Chinese Academy of Sciences, Shanghai, China) were used. Reagents included Dulbecco’s modified Eagle’s medium (DMEM; Gibco Inc.), fetal bovine serum (FBS; Gibco Inc.), trypsin, MTT powder and transwell chamber (Corning), N-cadherin, E-cadherin, and vimentin antibodies (Santa Cruz Biotechnology), matrix metalloproteinase (MMP)-2 and MMP-9 gelatinase kits (APPLYGEN, Beijing, China), and cytoskeletal staining kit (Millipore).

### Preparation of COE solution

Terpenoids from *Celastrus orbiculatus* (Batch No: 070510) were purchased from Professor Wang Qiang’s research group at the Traditional Chinese Medicine Institute of China Pharmaceutical University (Guangzhou, China) [7]. To extract terpenoids from *Celastrus orbiculatus*, the stem of *Celastrus orbiculatus* was cut, crushed into powder, and dried. Reflux extraction was repeated three times using 95 % ethanol. A rotary evaporator was used to recover the solvent to obtain the extract. The extract was added to diatomite, dried in a vacuum at low temperature, heated and refluxed with ethyl acetate, and filtered to obtain ethyl acetate extract. Terpenoids made up 68.3 % of the extract, and the rate of obtaining extract was approximately 2 %. Concentrated COE solution was prepared by hydrotropy of the extract using dimethyl sulfoxide and culture in serum-free medium. COE solution was filtered and sterilized at atmospheric pressure for later use.

### Instruments

Instruments included a 5 % CO_2_ incubator (Thermo Fisher Scientific, MA, USA), automatic enzyme-mark analyzer, protein electrophoresis chamber, power transfer device (Bio-Rad Company, CA, USA), SDS-polyacrylamide gel electrophoresis (SDS-PAGE) gel imaging analyzer (Bio-Rad Company), inverted fluorescence microscope, and confocal laser scanning microscope (CLSM; Olympus, Japan).

### Cell culture and passage

U251 and U87 cells were cultured with DMEM in a 5 % CO_2_ incubator at 37 °C. Cells were observed under an inverted fluorescence microscope. All cells used in this study were in the exponential phase.

### MTT assay of cell viability

DMEM containing 10 % FBS was used to prepare a single-cell suspension with a concentration of 3 × 10^5^ cells/mL. The suspension was placed in a 96-well plate with 100 μL/well. After attachment, cells were randomly divided into the control group and COE groups. Cells in COE groups were treated with different concentrations of COE ranging between 10 and 320 μg/mL, with five wells for each concentration. After cells were cultured in a 5 % CO_2_ incubator at 37 °C for 24, 48, or 72 h, 15 μL 3-(4,5-dimethyl-2-thiazolyl)-2,5-diphenyl-2-H-tetrazolium bromide (MTT) was added to each well in the dark. After 4 h, 100 μL dimethyl sulfoxide was added after the supernatant was discarded. The absorbance (*A*) value was read at 490 nm using a microplate reader. The experiment was repeated three times. The COE inhibition rate (%) was calculated as [1 - (*A* of cells in the COE group/*A* of cells in the control group)] × 100 %. The 50 % inhibitory concentration (IC_50_) was also calculated.

### Cell-matrix adhesion assay

Serum-free DMEM was used to dilute type I collagen stock solution to 10 μg/mL, which was placed in a 24-well plate overnight at 4 °C. Type I collagen was blocked for 1 h using 1 % bovine serum albumin and washed three times with phosphate-buffered saline (PBS). U87 and U251 cells in COE groups were treated with 20, 40, or 80 μg/mL COE for 24 h. Cells were starved overnight in serum-free DMEM, digested, centrifuged, and resuspended at a concentration of 3 × 10^5^ cells/mL. Cells were then plated at a concentration of 3 × 10^4^ cells/mL in the 24-well plate, with three wells for each concentration. Cells were cultured in a 5 % CO_2_ incubator at 37 °C for 1 h. The culture solution was then removed from the 24-well plate, and non-adherent cells were washed away three times with PBS. The remaining cells were fixed for 30 min with 2 % paraformaldehyde, stained with cresyl violet for 15 min, and observed under an inverted microscope. The experiment was repeated three times. Cell adhesion inhibition rate (%) was calculated as (1 – number of cells in the COE group/number of cells in the control group) × 100 %.

### Cell migration and invasion assays

For the wound-healing assay, U87 and U251 cells were cultured in DMEM at a concentration of 5 × 10^5^ cells/mL until cell confluence reached 90 %. Micropipette tips were used to make linear scratches, and the exfoliated cells were washed off three times with PBS. The remaining cells were starved overnight with serum-free medium to exclude the effect of proliferation on migration. Cells in COE groups were treated with 20, 40, or 80 μg/mL COE and cultured for another 24 h before images were taken. The experiment was repeated three times. The degree of wound healing (%), calculated as (scratch width of the control group - scratch width of the COE group)/scratch width of the control group × 100 %, was used to measure the migration capacity of cells.

In the transwell invasion assay, matrigel (1:8) was diluted with serum-free DMEM, and the basement membrane of the upper chamber of the transwell was coated. The solution was kept at 37 °C for 1 h to transform the matrigel aggregate into gel. Cells were prepared at a concentration of 5 × 10^5^ cells/mL in serum-free DMEM. Two hundred μL was added to the upper chamber of the transwell, and 600 μL culture medium containing 20 % FBS was added to the lower chamber. Cells in the COE group were treated with 20, 40, or 80 μg/mL COE, with three wells for each concentration. Cells at each concentration were cultured in a 24-well plate in a 5 % CO_2_ incubator at 37 °C for 24 h. The culture medium in each well was then discarded, and the chamber was washed twice with PBS. Cells that did not migrate were physically cleared from the upper chamber with cotton swabs. The cells that migrated were fixed with methanol for 15 min, stained with 0.1 % cresyl violet, washed three times with PBS, and air-dried. The chamber was inverted on a microslide and observed under a microscope. Five fields (200× magnification) were randomly selected for counting the number of migrated cells, and images were taken. In the transwell migration assay, matrigel was not used, otherwise the procedure was the same as that used in the invasion assay.

### E-cadherin, N-cadherin, and vimentin expression

U87 and U251 cells in COE groups were treated with 20, 40, or 80 μg/mL COE. After 24 h, total cellular protein was extracted. After bicinchoninic acid assay, SDS-PAGE was performed. Separated protein was transferred to a polyvinylidene fluoride membrane, kept at room temperature for 2 h with 5 % skim milk, and incubated at 4 °C overnight with the primary antibody (1:1000). The corresponding secondary antibody (1:1000) was added after membrane washing, and protein was incubated at room temperature for 2 h. ECL detection reagent was used to develop and detect specific protein bands.

### Gelatin zymography for detecting MMP-2 and MMP-9 activity

Supernatant was collected and cultured. After mixing with buffer solution at 1:1, 8 % SDS-PAGE containing 1 % gelatin was added, and electrophoresis was performed for 2 h. The gel was washed with 2.5 % Triton X-100 twice at room temperature for 30 min and transferred to substrate buffer (10 mmol/L Tris Base, 40 mmol/L Tris-Cl, 0.2 mol/L NaCl, 5 mmol/L CaCl_2_, 0.02 % Brij 35, pH 7.6). After reaction overnight at 37 °C and fixation for 2 h, Coomassie blue staining was performed for 20 min. Eluent destaining was performed until clear digestion bands were observed. Image analysis was performed on the electrophoresis results.

### Immunofluorescence and CLSM for detecting cytoskeletal organization

Cytoskeletal organization was visualized by using the actin cytoskeletal and focal adhesion staining kit (FAK100;Millipore,Billerica,MA). U87 and U251 cells were placed in a 6-well plate at a concentration of 5 × 10^5^ cells/mL. When cell confluence reached 50 %, cells were treated with 80 μg/mL COE for 24 h and then washed twice with PBS. Cells were fixed immediately with 4 % paraformaldehyde for 15 min at room temperature, and then permeabilized in 0.1 M PBS containing 0.2 % Triton X-100 for 5 min. After being blocked with 5 % bovine serum albumin, the cells were immune-labeled with anti-vinculin (1:200) at 37 °C for 1 h. Then, cells were incubated with FITC-anti-mouse (1:200) and treated with 0.5 μmol/L phalloidin-tetramethylrhodamine isothiocyanate (TRITC), placed in the chamber’s cassette for 45 min, then washed twice with PBS, stained with 6-diamidino-2-phenylindole (DAPI) for 5 min, and mounted with anti-fade fluorescence mounting medium. The structure of actin microfilaments and vinculin were evaluated by using confocal microscope (Olympus, Japan). All images were obtained under the same conditions of excitation and registration.

### Statistical analysis

Each experiment was repeated at least three times. Data were analyzed by one-way analysis of variance using SPSS 16.0 (SPSS Inc., Chicago, IL). Data are shown as mean ± standard deviation. *P*-values < 0.05 and *P*-values < 0.01 means difference were considered statistically significant.

## Results

### Effect of COE on viability of human glioblastoma cells

**Fig. 1:**
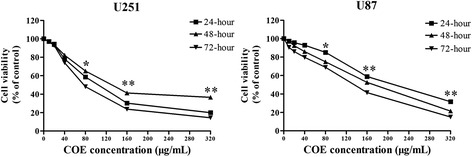
COE treatment inhibited the viability of U251 and U87 cells by the MTT assay. Cells were incubated with the indicated concentration of COE for 24 h. Dose- and time-dependent curve of inhibition rate of COE on U251 and U87 cells by the MTT assay. Date were presented as the means ± SD of three independent experiments performed in quintuplicate. *P < 0.05, **P < 0.01, as compared with the untreated control

Control U87 and U251 cells exhibited active growth in vitro, whereas cells treated with varying concentrations of COE for 24, 48, or 72 h showed significantly inhibited growth (Fig. 1; *P* < 0.01). The IC_50_ for U87 and U251 cells was 107.1 μg/mL and 186.4 μg/mL, respectively. Therefore, COE concentrations of 20, 40, and 80 μg/mL were used in subsequent experiments to exclude the cytotoxic effect of COE on cell adhesion, invasion, and migration.

### Effect of COE on adhesion of human glioblastoma cells

**Fig. 2:**
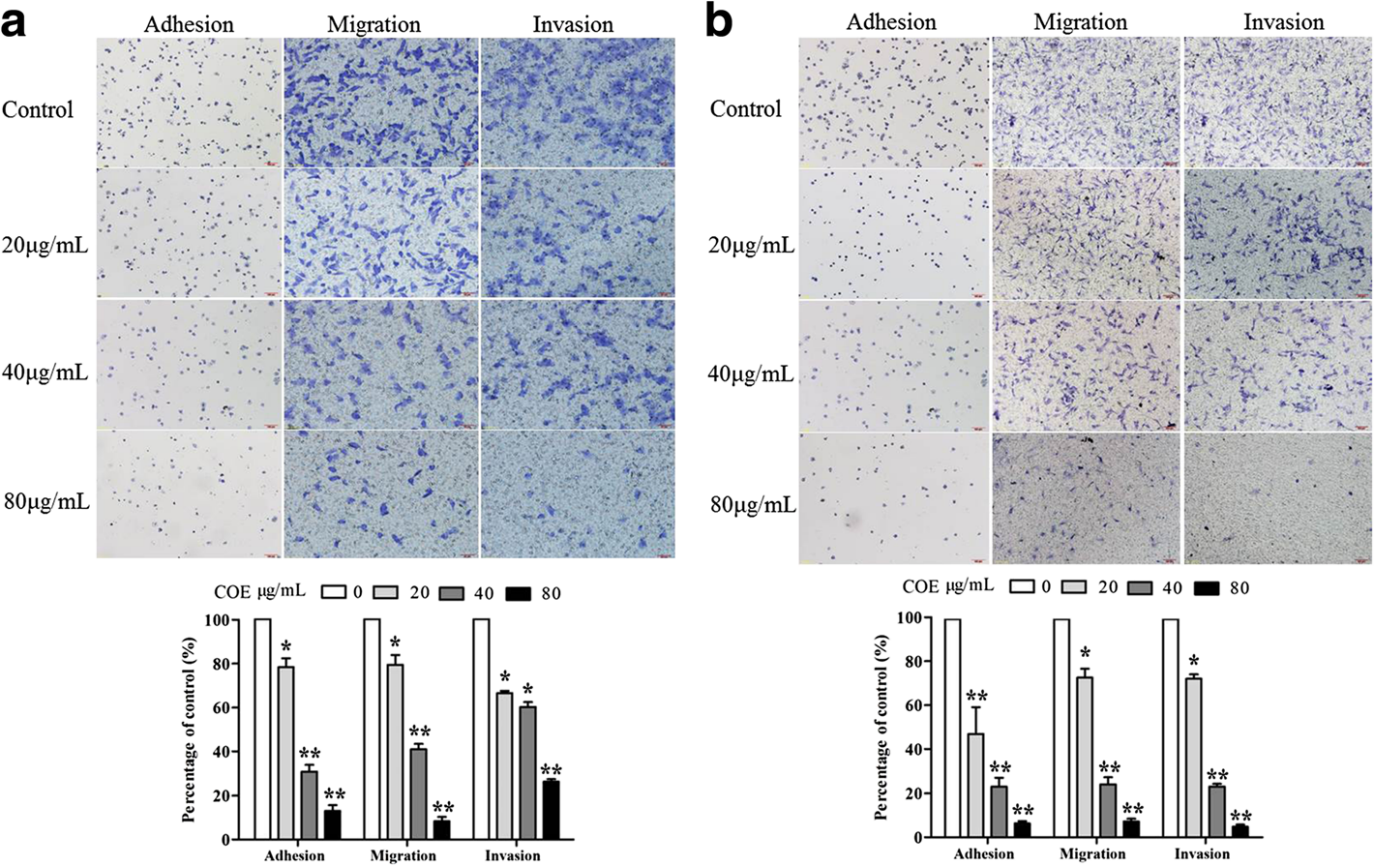
COE treatment for 24 h inhibited the adhesion, invasion and migration of (**a**) U87 and (**b**) U251 cells in a dose-dependent manner. Cell migration and invasion were assessed after 24 h incubation by transwell assay. The cell adhesion ability was performed by the cell adhesion assay. Top: cresyl violet staining (200× magnification). Bottom: quantification data. Values are expressed as means ± SD of three independent experiments.*P < 0.05, **P < 0.01, as compared with the untreated control

The number of adherent U87 and U251 cells in type I collagen-containing extracellular matrix (ECM) was observed after COE treatment for 24 h. COE treatment significantly reduced cell adhesion in a dose-dependent manner (Fig. 2a-b, *P < 0.05 **P < 0.01).

### Effect of COE on invasion and migration of human glioblastoma cells

**Fig. 3:**
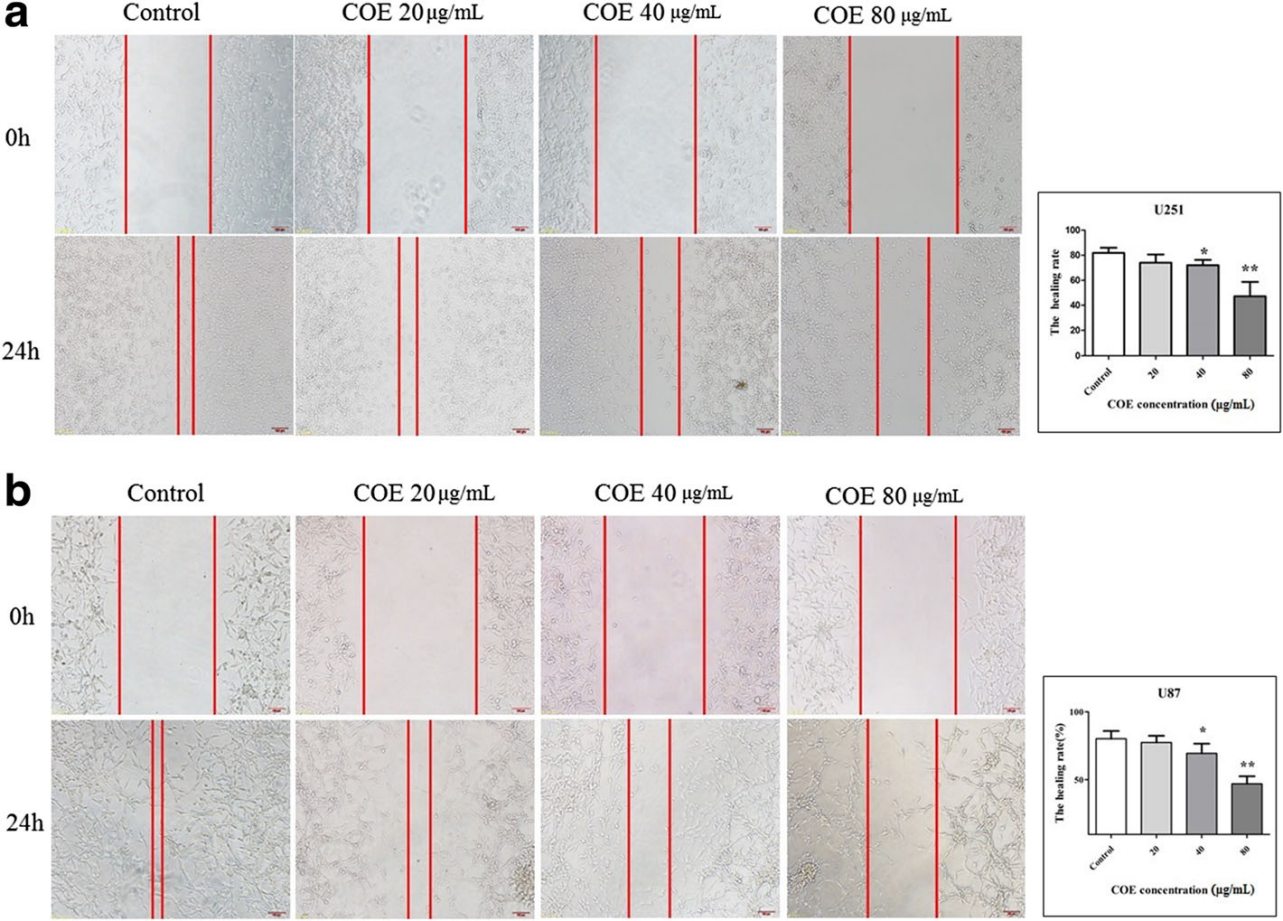
COE treatment for 24 h inhibited the migration of (**a**) U87 and (**b**) U251 cells in a wound-healing assay in a dose-dependent manner. Left: images of wound-healing assay (200× magnification). Right: quantification data. *P < 0.05, **P < 0.01, as compared to controls

In the wound-healing assay, COE treatment for 24 h significantly inhibited the migration of U87 and U251 cells in a dose-dependent manner (Fig. 3a-b, *P < 0.05 **P < 0.01). Furthermore, in the transwell migration and invasion assay, COE treatment significantly and dose-dependently decreased the number of transmembrane cells (Fig. 2a-b, *P < 0.05 **P < 0.01), indicating that COE inhibited cell migration and invasion.

### Effect of COE on expression of epithelial-mesenchymal transition (EMT)-related proteins and MMPs in human glioblastoma cells

**Fig. 4:**
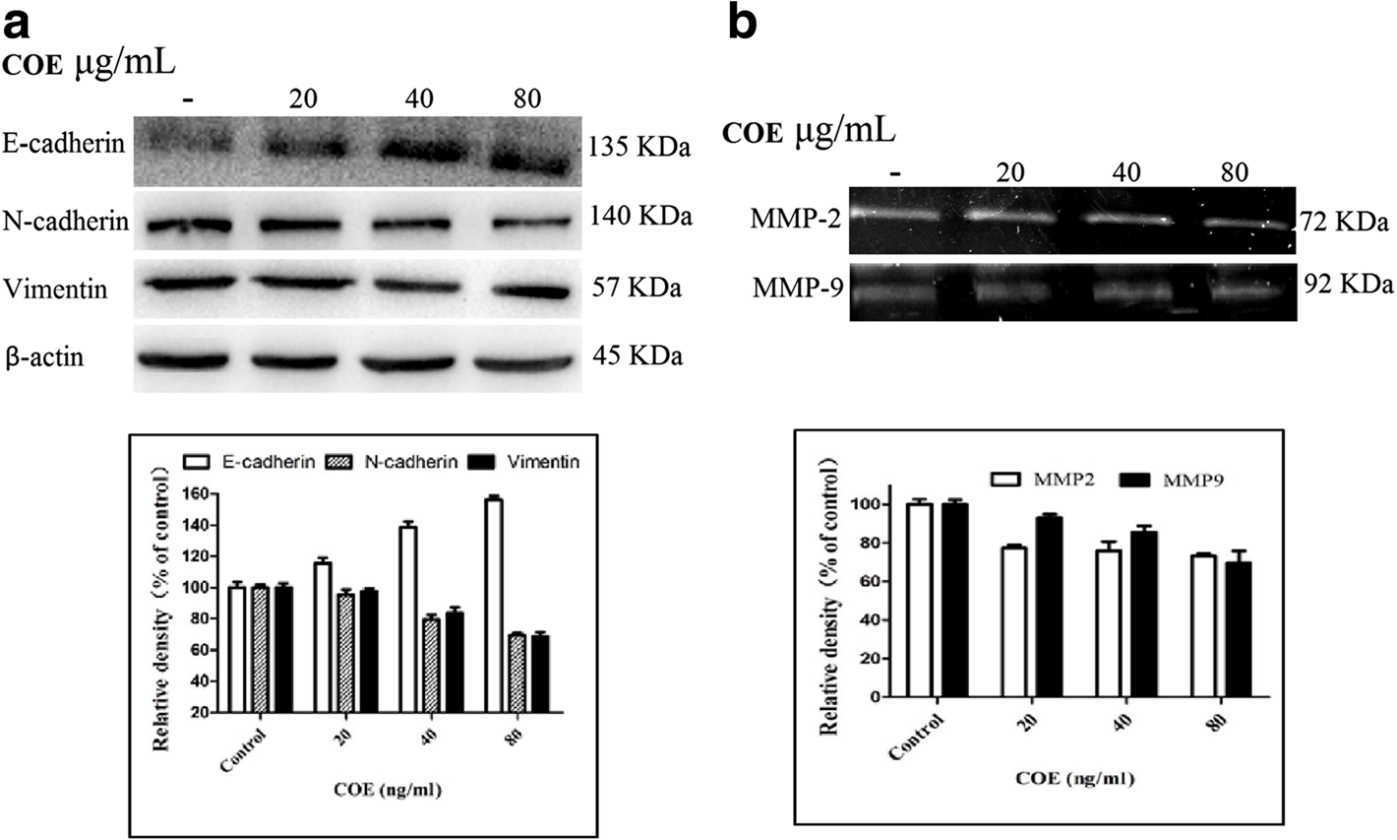
COE treatment U251 cells for 24 h altered the expression of EMT-related proteins and enzymes in a dose-dependent manner. **a**: vimentin, E-cadherin, and N-cadherin expression as shown using western blots. Bottom: quantification data. **b**: MMP-2 and MMP-9 expression as shown using gelatin zymography. Bottom: quantification data. *P < 0.05 **P < 0.01, as compared with the untreated control

U87 and U251 cells treated with COE for 24 h showed increased expression of E-cadherin and decreased expression of N-cadherin and vimentin in a dose-dependent manner (Fig. 4). Furthermore, COE treatment dose-dependently decreased MMP-2 and MMP-9 expression.

### Effect of COE on cytoskeletal organization in human glioblastoma cells

**Fig. 5:**
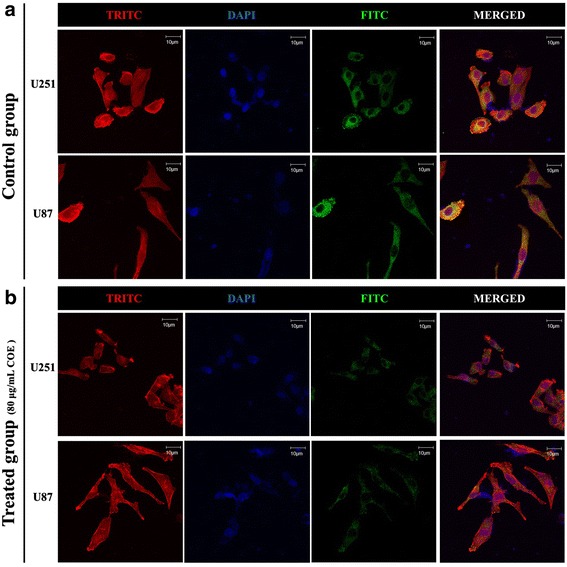
COE treatment induced cytoskeletal collapse. The organization of the filamentous actin (F-actin, red fluorescence), the distribution of vinculin (green fluorescence), and the nuclear (blue fluorescence) were observed using confocal fluorescence microscopy (magnification, ×1000; scale bar, 10 μm).**a** U251 and U87 cells of control group. **b** 80 μg/mL COE treated U251 cells

As illustrated by confocal microscope, both U251 and U87 cells were immunopositive to F-actin (red) and vinculin (green). Treatment with 80 μg/mL COE for 24 h resulted in a significant collapse in cytoskeletal cytoskeletal organization and reduction in the distribution of vinculin compared with control group (Fig. 5). After treatment with COE, actin broke progressively, and the control group maintained with stable and ordered structure.

## Discussion

Gliomas are common intracranial tumors. Gliomas demonstrate invasive growth and exhibit ill-defined pathological features, and due to their growth in areas of the brain that serve important functions, they cannot be completely removed by surgery [8]. The common invasion route of glioblastomas is along basement membranes and medullated fibers.

Invasion and migration are the main biological characteristics associated with tumor malignancy [9]. Liotta et al. proposed a three-step process of invasion by malignant tumors: tumor cells attach to the ECM, degrade ECM via proteolytic enzymes, and migrate into the degraded matrix [10]. This process can explain how glioma cells invade adjacent brain tissues in a single-cell manner, grow invasively along basement membranes and white matter fibers, rarely exhibit blood vessel or lymphatic metastasis, and lead to poor prognosis and patient survival rates [11]. Thus, it is of great significance to control the malignant invasion of glioma cells and thereby improve patient outcomes.

COE is the product isolated form the plant of *Celastrus orbiculatus,* a Chinese traditional herb. We found that the main effective ingredients extracted from COE, namely diterpenoids, triterpenoids, and alkaloids, inhibited tumor cell activity. Our previous studies demonstrate that COE exerts cytotoxic effects, induces apoptosis, and inhibits tumor cell adhesion and migration [12, 13]. Nevertheless, the most effective chemical which is exerting the anti-cancer function in COE is not clear, more research was need to find the biologically active components and corresponding function. In the present study, we examined the effects of COE on human glioblastoma U87 and U251 cell lines using various molecular biology methods to explore the mechanisms by which COE acts against human gliomas.

We used the MTT assay to assess cell viability and an ECM adhesion assay to assess cell adhesion, which may reflect the potential for cell migration resulting from an interaction between ECM forces and the movement of tumor cells. After treatment with COE U87 and U251 cells showed decreased growth and adhesion depending on the concentration and duration of treatment. These results indicate that COE could inhibit cell viability, ECM adhesion, and tumor cell metastases.

Glioblastoma invasion is a complex process that is a major contributor to poor prognosis. ECM degradation appears to be the most important feature of this process [14]. The ECM undergoes continuous remodeling in active tissues, during which many proteases, including MMPs, participate in the destruction of normal brain tissue. MMPs, which are cation-dependent endopeptidases with high homology, degrade most components of the ECM [15]. Due to their broad distribution in the human body and wide range of hydrolysis substrates, MMP-2 and MMP-9 are regarded as key enzymes for hydrolyzing the ECM and promoting tumor invasion. To explore whether COE could inhibit ECM degradation, we used gelatin zymography to observe MMP-2 and MMP-9 expression in U87 and U251 cells. Treatment with COE for 24 h decreased MMP-2 and MMP-9 expression. Thus, COE may inhibit MMP-2 and MMP-9 activity and ECM hydrolysis, thereby attenuating the invasion and migration of glioblastoma cells. Indeed, we also found thatCOE inhibited the invasion and migration of glioblastoma cells in various migration and invasion assays.

To further explore the molecular mechanism by which COE inhibits the invasion and migration of U87 and U251 cells, we assessed the levels of E-cadherin, N-cadherin, and vimentin, which serve different functions in invasion and migration. E-cadherin, which is widely distributed in human epithelial cells and is a member of the calcium-dependent family of adhesion molecules, mediates intercellular adhesion to maintain the proper morphology and polarity of tissues, and participates in intracellular signal transduction [16]. Intercellular adhesion declines when E-cadherin expression decreases or is absent, and the removal of E-cadherin from primary lesions promotes cell invasion and migration [17]. By contrast, decreased levels of N-cadherin indicate the recovery of cell polarity and result in the inhibition of tumor cell invasion and migration [18]. Also, decreased levels of vimentin, are associated with decreased migration and invasion of tumor cells [19]. Thus, changes in levels of E-cadherin, N-cadherin, and vimentin can serve as indicators of EMT abnormalities [20]. In the present study, we found that COE treatment increased E-cadherin expression and decreased N-cadherin and vimentin expression, indicating that COE may inhibit the process of EMT and the invasion and migration of glioblastoma cells.

Increased cell motility via cytoskeletal remodeling promotes tumor cell invasion and migration [19, 21]. The cytoskeletal usually connects to the cytomembrane, influences interactions between the membrane and ECM, and controls the activity of membrane receptors and adhesion plaques of microfilaments to regulate cell movement and adhesion [15, 16]. F-actin stress bundles (polymerized f-actin filaments) and focal adhesions are associated with cell migration and adhesion. Therefore, we evaluated them by using TRITC-phalloidin fluorescence intensity and FITC-vinculin, respectively. After treatment with COE, the F-actin broke progressively, and the focal adhesions declined dramatically compared with control group. The results indicate that COE had changes in the human glioblastoma cells cytoskeletal structure along with focal adhesions, thereby inhibiting cell migration and adhesion.

## Conclusions

COE has dose-dependent effects on cell viability, adhesion, migration, and invasion, as well as the expression of EMT-related proteins and MMPs in human glioblastoma U87 and U251 cells. By inhibiting cell viability and the process of EMT and regulating cytoskeletal organization, COE could attenuate the adhesion, migration, and invasion of human glioblastoma cells.

## Abbreviations

COE, celastrus orbiculatus extract; ECM, extracellular matrix; EMT, epithelial-mesenchymal transition; MMPs, matrix metalloproteinases; TRITC, phalloidin-tetramethylrhodamine isothiocyanate

## References

[1] Trabelsi S, Brahim DH, Ladib M, Mama N, Harrabi I, Tlili K, Yacoubi MT, Krifa H, Hmissa S, Saad A, Mokni M (2014). Glioma epidemiology in the central Tunisian population: 1993-2012. Asian Pac J Cancer Prev.

[2] Back MF, Ang EL, Ng WH, See SJ, Lim CC, Tay LL (2007). Improvements in quality of care resulting from multidisciplinary tumour clinic in the management of high-grade glioma. Ann Acad Med Singapore.

[3] Demuth T, Rennert JL, Hoelzinger DB, Reavie LB, Nakada M, Beaudry C (2008). Glioma cells on the run-the migratory transcriptome of 10 human glioma cell lines. BMC Genomics.

[4] Wang M, Zhang X, Xiong X, Yang Z, Sun Y, Yang Z (2012). Efficacy of the Chinese traditional medicinal herb Celastrus orbiculatus Thunb on human hepatocellular carcinoma in an orthothopic fluorescent nude mouse model. Anticancer Res.

[5] Wang W, Liu Y, Dai X (2010). Apoptosis of gastric cancer SGC-7901 cells induced by Celastrus orbiculatus Thunb extract and mechanism of induction. Chin J Biologicals.

[6] Ma H, Qian Y, Zhang H (2013). Celastrus orbiculatus extract could inhibit human colorectal carcinoma HT-29 cells metastasis via suppression of the mTOR signaling pathway. Life Science Journal.

[7] Qian YY, Zhang H, Hou Y, Yuan L, Li GQ, Guo SY (2012). Celastrus orbiculatus extract inhibits tumor angiogenesis by targeting vascular endothelial growth factor signaling pathway and shows potent antitumor activity in hepatocarcinomas in Vitro and in Vivo. Chin J Integr Med.

[8] Walker C, Baborie A, Crooks D, Wilkins S (2011). M D Jenkinson. Biology, genetics and imaging of gial cell tumor.J. Br J Radiol.

[9] Gassmann P, Enns A, Haier J (2004). Role of tumor cell adhesion and migration in organ-specific metastasis formation. Onkologie.

[10] Liotta LA (1986). Tumor invasion and metastasis role of the extra cellular matrix: Rhoads Memorial Award lecture. Cancer Res.

[11] Wilkes G, Hartshorn K (2009). CoIon, rectal, and anal cancers. Semin Oncoi Nurs.

[12] Zhu YD, Liu YQ, Qian YY, Dai XJ, Yang L, Chen J (2014). Research on the efficacy of Celastrus Orbiculatus in suppressing TGF-β1-induced epithelial-mesenchymal transition by inhibiting HSP27 and TNF-α-induced NF-k B/Snail signaling pathway in human gastric adenocarcinoma. BMC Comlement Altern Med.

[13] Zhu YD, Liu YQ, Qian YY, Zhan H, Li GQ, Yang L. Extracts of Celastrus orbiculatus exhibit anti-proliferative and anti-invasive effects on human gastric adenocarcinoma cells. Chin J Integr Med. 2014; Epub ahead of print 10.1007/s11655-014-1951-y 25382615

[14] Xie Q, Mittal S, Berens ME (2014). Targeting adaptive glioblastoma: an overview of proliferation and invasion. J Neuro Oncology.

[15] Nathoo N, Chahlavi A, Barnett GH, Toms SA (2005). Pathobiology of brain metastase. J Clin Pathol.

[16] Blaschuk OW, Devemy E (2009). Cadherins as novel targets for anti-cancer therapy. Eur J Pharmacol.

[17] Gloushankova NA (2008). Changes in regulation of cell-cell adhesion during tumor transformation. Biochemistry.

[18] Camand E, Peglion F, Osmani N, Sanson M, Etienne-Manneville S (2012). N-Cadherin expression level modulates integrin-mediated polarity and strongly impacts on the speed and directionality of glial cell migration. J Cell Sci.

[19] Akakura S, Gelman IH (2012). Pivotal role of AKAP12 in the regulation of cellular adhesion dynamics: control of cytoskeletal architecture, cell migration, and mitogenic signaling. J Signal Transduct.

[20] Mousumi T, Khan Md A, Fu J (2014). Epithelial to mesenchymal transition inducing transcription factors and metastatic cancer. J Tumour Biol.

[21] Hotulainen P, Lappalainen P (2006). Stress fibers are generated by two distinct actin assembly mechanisms in motile cells. J Cell Biol.

